# Granulocyte Colony Stimulating Factor Adjuvant Role on the Immunological Response to Hepatitis B Vaccine in Patients With Cirrhosis: A Double Blind Randomized Placebo Controlled Trial

**DOI:** 10.5812/hepatmon.15447

**Published:** 2014-05-07

**Authors:** Kamran Bagheri Lankarani, Mozaffar Talebzadeh, Ahad Eshraghian, Seyed Ali Malek-Hosseini

**Affiliations:** 1 Health Policy Research Center, Shiraz University of Medical Sciences, Shiraz, IR Iran; 2 Department of Internal Medicine, Shiraz University of Medical Sciences, Shiraz, IR Iran; 3 Organ Transplant Research Center, Shiraz University of Medical Sciences, Shiraz, IR Iran

**Keywords:** Hepatitis B, Granulocyte Colony-Stimulating Factor, HBV Vaccine, Liver Cirrhosis, Clinical Trial

## Abstract

**Background:**

Patients with liver cirrhosis have usually poor antibody response to hepatitis B virus (HBV) vaccination.

**Objectives:**

This study aimed to investigate the effect of granulocyte colony stimulating factor (G-CSF) on increasing antibody titers, after HBV vaccination, in patients with liver cirrhosis waiting for transplantation.

**Patients and Methods:**

From 56 patients with cirrhosis, 28 patients were allocated to receive double dose HBV vaccine (40 μgr) plus G-CSF and 28 patients were allocated to receive double dose HBV vaccine (40 μgr) plus placebo. Injections were performed on weeks 0, 4 and 8 and the blood samples were obtained one month after each vaccination session.

**Results:**

There was no statistically significant difference between anti-HBV antibody titers in patients receiving double dose HBV vaccination plus G-CSF and patients receiving double dose HBV vaccination plus placebo, after first, second or third vaccination rounds (P > 0.05). Although the adjuvant G-CSF injection did not cause significant increased antibody titers in our patients compared to the placebo group, the increase in antibody titers following vaccination, happened faster in this group, compared to the placebo group.

**Conclusions:**

The present study showed that G-CSF is not superior to placebo in production of protective antibody titers after HBV vaccination but could result in a more rapid antibody response, compared to the placebo.

## 1. Background

Liver transplantation is now the only treatment for long term survival of patients with advanced liver diseases and is now widely performed worldwide (1). Improvements in diagnostic methods and treatment options have led to a significant improvement in the survival of patients with cirrhosis, which results in expansion of the transplant waiting lists.

Patients undergoing liver transplantation receive several immunosuppressive medications, making them susceptible to numerous infections in the post-transplant period. Hepatitis B virus (HBV) infection is one of the infections that may occur, even in patients without any evidence of HBV infection before transplantation (2). Massive transfusions of blood products and receiving organ from anti-HBC positive donors have been associated with HBV infection in the post-transplantation period (3). Therefore, HBV vaccination is recommended for all patients with cirrhosis, before transplantation (4). Several studies have been conducted, investigating the effectiveness of HBV vaccination in patients with cirrhosis, without desirable results (5, 6). Protective antibody titers have only been detected in 16-28% of vaccinated patients (7) and after duplication of the vaccine dosage 37% of patients had protective antibody titers (8). Old age, underlying liver disease and its severity and decreased humoral and cellular immune responses in cirrhosis have been suggested as reasons for the poor response to HBV vaccination in patients with cirrhosis (8). HLA-DR 3 and HLA-DR 7 are known to be involved in the immunological response to HBV vaccination in the general population. Patients with these certain types of HLA have problems in HBS antigen presentation to T-cells, contributed to poor antibody response (8, 9). Therefore the routine vaccination and even double-dose vaccines are ineffective in patients with liver cirrhosis and another strategy should be utilized to increase antibody response to vaccination. Granulocyte monocyte colony stimulating factor (GM-CSF) or granulocyte colony stimulating factor (G-CSF) has been utilized as an adjunct therapy to increase the effectiveness of HBV vaccination, especially in patients with end stage renal disease (10).

## 2. Objectives

This study aimed to investigate the effect of G-CSF on increasing antibody titers, after HBV vaccination, in patients with liver cirrhosis waiting for transplantation.

## 3. Patients and Methods

### 3.1. Study Design and Patients

We undertook a prospective, double blind, randomized, placebo-controlled clinical trial between June 2011 and June 2012 in Shiraz University of Medical Sciences, Shiraz, Iran. Eligible participants were male and female patients with non HBV-induced liver cirrhosis, aged 20-65 years old, who were referred from hepatology clinics to be included in the transplantation waiting list at Shiraz University of Medical Sciences, Shiraz, Iran. We included HBS Ag negative, HBS Ab negative and HBC Ab negative patients with Child score B and higher and MELD score 15 or higher. Patients with renal failure, history of hepatocellular carcinoma and cholangiocarcinoma, hypersensitivity to G-CSF and patients receiving immunosuppressive medications like tacrolimus, cyclosporine, azathioprine and sirolimus were also excluded. Patients developing drug reactions during the study were also excluded.

### 3.2. Randomization and Masking

We randomly allocated eligible participants in one to one ratio to receive HBV vaccine plus G-CSF or HBV vaccine plus placebo. Randomization was performed by a statistician without any clinical involvement in the study, using blocked randomization with fixed block sizes of four and random digit tables. Patients, investigators who gave the treatment, those assessing outcomes and those who performed the statistical analysis were masked to the allocation. G-CSF and placebo were provided from the same pharmaceutical company (Pooyesh Darou, Tehran, Iran), in identical packages and syringes to ensure that they are indistinguishable by the patients and investigators.

### 3.3. Procedures

After randomization, each patient in G-CSF group had two injections: an expert nurse injected a double dose HBV vaccine (40 μgr) in the left deltoid muscle and simultaneous G-CSF ampoule (300 μgr) subcutaneously on the right arm. Each patient in the placebo group also had two injections: double dose HBV vaccine (40 μgr) in the left deltoid muscle and simultaneous placebo ampoule (1cc) subcutaneously on the right arm were injected. All patients were observed for two hours regarding probable reactions. Injections were repeated one month and two months following the first injection (0, 4 and 8 weeks). Before second and third injections (in fourth and eighth weeks) 5 mL blood samples were obtained from each patient and after separating the plasma, were maintained in a -70ºC refrigerator. One month after the last injection (in the 12th week) another blood sample was obtained from each patient and kept in the same circumstances. Primary outcome was defined as an antibody titer above 10 mIU/mL. HBV antibody level was measured according to manufacturers’ instructions, using ELISA kits (DiaSorin diagnostic kits, Italy). HBV vaccines were obtained from Pasture Institute, Tehran, Iran. The study was registered in Australian New Zealand Clinical Trial Registry (ANCTR) with number ACTRN12611001180909.

### 3.4. Statistical Analysis

Primary outcome of the study was defined as level of antibody against hepatitis B surface antigen (anti-HBS antibody) with 10 IU/L or more after blood analysis. Primary findings were assessed after the first, second and third injections. All variables were expressed as the mean ± SD. The differences between the cases and control group were tested statistically with the Chi-square test and Pearson correlation index. Baseline data with normal distribution were compared between the two groups, using independent-samples t test. Baseline data with skewed distribution were compared using Mann-Whitney U test. Categorical data were compared using χ2 or Fisher exact tests. For primary outcome a P value of < 0.05 was considered statistically significant. SPSS software 2010 (Tokyo, Japan) was used.

### 3.5. Ethics and Consent

Written informed consents, consisting of all study steps and the possible risks and benefits were signed by all the patients after careful discussion. The study protocol was conducted in accordance with the Helsinki Declaration, as revised in Edinburgh, 2000. The study protocol was also approved by the Ethical Committee of Shiraz University of Medical Sciences.

## 4. Results

**Figure 1.**
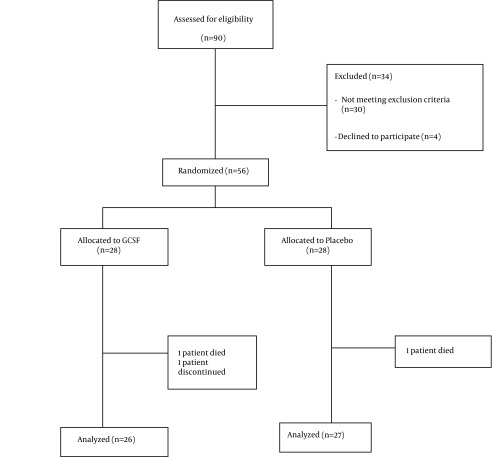
Flow Diagram of Trial

**Figure 2.**
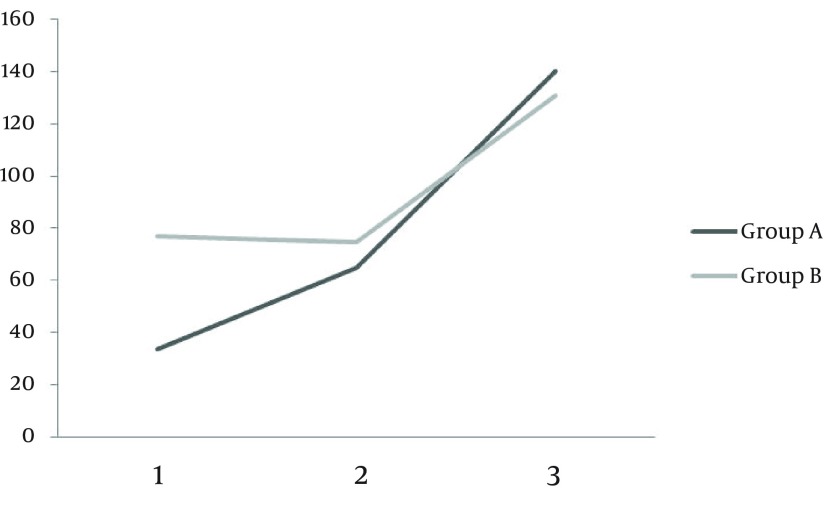
Diagram Showing an Increase in Antibody Titers Following Vaccination

Among 90 patients evaluated, 56 patients were eligible and included in the study. Twenty eight patients were randomly assigned to receive double dose HBV vaccine plus G-CSF (group A) and the other 28 received double dose of HBV vaccine plus placebo (group B). During the study two patients were excluded from the G-CSF group (one died and another one lost follow up) and one patient was excluded from the placebo group due to discontinuation of placebo usage (Figure 1). Baseline characteristics of patients are outlined in Table 1. No statistically significant differences were observed in terms of baseline characteristics between placebo and G-CSF groups. Serum anti-HBV antibody titers after first, second and third vaccination rounds in both groups are demonstrated in Table 2. There was no statistically significant difference between HBV antibody titers, in patients receiving double dose HBV vaccination plus G-CSF and patients receiving double dose HBV vaccination plus placebo after first, second and third vaccination rounds (P > 0.05). Mean antibody titer difference after first, second and third vaccinations were not statistically significant between the two groups (Table 3). Although an adjuvant G-CSF injection was not able to increase antibody titers in our patients compared to the placebo group, a more rapid increase in antibody titers was observed in these patients following vaccination (Figure 2).

There were no serious adverse events observed in patients during this trial convincing them to quit the study.

**Table 1.** Baseline Characteristics of the Patients ^a^

	HBV Vaccine + Placebo	HBV Vaccine + G-CSF	P Value
**Number**	28	28	
**Age, y**	36.91 ± 12.54	39.17 ± 16.5	NS
**Sex, **m**ale/**f**emale**	15/13	14/14	NS
**Causes of liver cirrhosis**			
Cryptogenic	14	13	
PSC	2	3	
HCV	3	1	
AIH	4	7	
Budd-chiari	3	3	
Wilson	2	1	
**Duration of **c**irrhosis **f**rom **d**iagnosis, mo**			NS
< 6	19	10	
6-12	4	11	
>12	5	7	
Ascites, +/-	15/3	17/11	
**Child **s**core**			NS
7-9	23	22	
> 9	5	6	
**MELD score**			NS
15	4	5	
15-19	18	16	
≥ 20	6	7	
**BMI, kg/m** ^**2**^			NS
< 20	3	1	
20-24	13	15	
25-39	9	10	
> 30	3	2	

^a^ Abbreviations: PSC; Primary Sclerosing Cholangitis, HCV; Hepatitis C Virus, AIH; Autoimmune Hepatitis, BMI; Body Mass Index, NS; Not Significant.

**Table 2.** The Effect of G-CSF on anti HBV Antibody Titers After Each Round of HBV Vaccination

	G-CSF, No. (%)	Placebo, No. (%)	Total, No. (%)	P Value
**First round**				0.16
Ab titers ≤ 10 (%)	20 (76.9)	16 (59.3)	36 (67.9)	
Ab titers > 10 (%)	6 (23.1)	11 (40.7)	17 (32.1)	
**Second round**				0.49
Ab titers ≤ 10 (%)	14 (53.8)	12 (44.4)	26 (49)	
Ab titers > 10 (%)	12 (46.2)	15 (55.6)	27 (51)	
**Third time**				0.30
Ab titers ≤ 10 (%)	3 (11.5)	6 (22.2)	9 (16.9)	
Ab titers > 10 (%)	23 (88.5)	21 (77.8)	44 (83.1)	

**Table 3.** Mean HBV Antibody Titers in Two Groups, Following First, Second and Third Sessions of Vaccinations ^a^

	First Time	Second Time	Third Time
**Group A, mIu/mL**	33.5 ± 8.52	65.19 ± 10.84	165.09 ± 21.05
**Group B, mIu/mL**	76.59 ± 11.06	74.44 ± 10.69	130.05 ±12.16
**P value**	0.16	0.9	0.45

^a^ Data are presented as Mean ± SD.

## 5. Discussion

Although G-CSF has been utilized in different studies to enhance the effect of HBV vaccination in immune response induction, as an adjuvant therapy, our study is the first one using G-CSF as an adjunct modality with HBV vaccination in patients with liver cirrhosis. Our results did not show any significant difference between patients receiving G-CSF and patients receiving placebo in terms of increase in the antibody titers. However, a more rapid rise in antibody titers was observed in patients treated with G-CSF, after all three sessions of vaccinations. Based on our findings, protective titers of HBV antibody (> 10 Iu/mL) was obtained in only 32% of our patients even with double dose vaccination (with and without G-CSF). This effect was increased to 50.1% and 83% after second and third vaccination sessions, respectively. We suggest a complete three session HBV vaccination in patients with liver cirrhosis since one or two vaccination sessions (even with double dose) failed to produce protective antibody titers in these patients.

Previous studies showed that the usual dose (20 μgr) of recombinant HBV vaccine was not efficient for producing protective antibody titers in patients with liver cirrhosis (5, 6). Therefore, in some other studies double dose HBV vaccines were used to increase the potency of immunologic responses (11, 12). A prospective study by Horlander et al. showed that HBV vaccine dose duplication could result in better antibody responses, compared to single dose vaccination, but the final response was still very low (7). Another prospective study among patients with cirrhosis in transplant waiting list showed that double dose of HBV vaccine was not capable of producing protective antibody titers in majority of patients on the transplantation day and one and two years after liver transplantation (8). A preliminary prospective trial using double dose HBV vaccine had similar results (9). Therefore, another strategy rather than doubling the dose of the vaccine should be sought to overcome the problem.

Colony stimulating factors (CSFs) are a group of multi-potential hematopoietic cell growth factors which have been widely used in clinical settings (13). CSFs in the form of GM-CSFs have been utilized in several studies as adjuvants to HBV vaccination in patients with end stage renal diseases and were helpful to induce better antibody responses in these patients (14). Therefore, CSFs can be used in patients with cirrhosis in transplant waiting list to induce immune response after HBV vaccination. G-CSF is a cytokine produced by many kinds of cells like endothelial cells, macrophages and lymphocytes and has been used for recovery of neutropenia after chemotherapy and increasing stem cells in circulation (15). This cytokine can increase neutrophil count, improve antigen presenting cell (APC) function and is involved in immune system development (16). G-CSF also mobilizes T helper 2-inducing dendritic cells (17), which can induce specific immune responses against HBV (18). In the present study the effect of G-CSF on increasing antibody titers following double dose HBV vaccination has been investigated. Previous studies regarding efficacy of HBV vaccination in patients with cirrhosis are mainly retrospective or prospective cohort. Our study is the first placebo controlled trial about efficacy of double dose HBV vaccination in patients with cirrhosis. Our study is unique because it is the first study using G-CSF as an adjunct modality to HBV vaccination in patients with cirrhosis. However, the results about ineffectiveness of G-CSF in immune response induction should be interpreted cautiously, due to relatively small sample size of this study. Finally we must emphasize on the point that patients receiving G-CSF had more rapid antibody response to HBV vaccination. Future prospective trials with larger sample sizes are necessary to further elucidate this issue.
